# Real-World Experience of Clinical Outcomes of Microscopic Margin Positivity After Radical Gastrectomy from a Tertiary Cancer Center in Northeast India

**DOI:** 10.1007/s13193-024-02081-y

**Published:** 2024-09-07

**Authors:** Akash Guha, Ashutosh Sahewalla, Dilip Killing, Manthan Thakkar, Gaurav Das, Deep Jyoti Kalita, Abhijit Talukdar

**Affiliations:** Department of Surgical Oncology, Dr B Borooah Cancer Institute, Room No. 30, Surgical OPD, Gopinathnagar, Guwahati, Assam 781016 India

**Keywords:** Positive margin, Gastric cancer, Gastrectomy, R1 resection

## Abstract

Surgical resection for gastric adenocarcinoma (GAC) remains the only potentially curative treatment, and the use of neoadjuvant and adjuvant therapy improves survival in patients with advanced gastric cancer. Margin-positive resection is a known poor prognostic factor. A retrospective observational study of patients undergoing radical gastrectomy of any type for GAC was done at a tertiary care cancer center in Northeast India. The study included patients who were operated on from 1 January 2017 to 31 December 2021 (5 years), and they were followed up to 31 March 2024. A total of 172 patients underwent gastrectomy of any type for GAC during the study period of which 13 patients were found to have microscopic positive (R1) histopathological margin (7.6%). The median age of the patients with positive margins was 48 years (range 27 to 69 years). The male-to-female ratio was 9:4. Ten patients (77%) had poorly differentiated or signet-ring cell carcinoma. The distal margin was the most frequent margin which was positive (84.6%). Neoadjuvant chemotherapy was used in only 23.1% patients. At the end of our study period, only 1 patient out of 13 patients was alive. Median disease-free survival (DFS) was 16.2 months (95% confidence interval 1.2 to 31.1 months). Median overall survival (OS) was 20.2 months (95% confidence interval 9.3 to 31.2 months). Patients who have microscopic positive margins after gastrectomy are found to have a high incidence of poorly differentiated or signet-ring cell carcinoma.

## Introduction

Gastric adenocarcinoma (GAC) is the fifth most frequently diagnosed cancer and the fifth leading cause of cancer-related death worldwide [1]. The incidence of gastric cancer in the Kamrup Urban population–based cancer registry (PBCR) is 6.3% [2]. The PBCR saw an annual percentage increase of 6.5% among males and 11.7% among females in the incidence of gastric cancer. Surgical resection remains the only potentially curative treatment for GAC, and the use of neoadjuvant and adjuvant treatment improves survival in advanced gastric cancer. Margin-positive resection is a known poor prognostic factor.

The National Comprehensive Cancer Network (NCCN) describes a negative margin (R0) as the absence of cancer at resection margins, whereas R1 and R2 are terminologies used for the presence of microscopic and macroscopic cancer at the resection margins, respectively. The Japanese Gastric Cancer Association has recommended a gross proximal margin of at least 2 cm for a T1 cancer, at least 3 cm for a T2 or deeper cancer with Bormann types 1 and 2 growth pattern, and at least 5 cm for cancer with an infiltrative growth pattern (Bormann types 3 and 4) [3]. Recent ESMO guidelines recommend a 3 cm margin for intestinal-type gastric carcinoma and 5 cm for diffuse-type GC. These recommendations have been made to decrease the chances of having a microscopic positive margin. However, the submucosal invasion may extend far from the macroscopic tumor margin, especially the diffuse type, which leads to a positive final margin [4].

The incidence of positive margin ranges between 0.9 and 59% despite improvements in surgical technique and the use of intra-operative frozen section examination [5].

The study aims to estimate the clinicopathological characteristics of the patients and their tumors in which the margins were microscopically positive after radical gastrectomy.

## Materials and Methods

This is a retrospective, observational study done in a tertiary care cancer center in Northeast India. The study included patients who were operated on from 1 January 2017 to 31 December 2021 (5 years). All the patients with a proven histological diagnosis of gastric adenocarcinoma (GAC) and who underwent curative intent gastrectomy of any type were identified from the register of major surgical operations. The final histopathology reports were checked, and the cohort of patients who had microscopic positive surgical margins (R1) was identified and included for analysis. The medical records of these patients, both physical files and electronic medical records (EMR), were comprehensively reviewed. The hospital-based cancer registry (HBCR) data was used to gather follow-up data on these patients till 31 March 2024. Data obtained were recorded in a proforma with details including a socio-demographic profile of the patient, tumor characteristics, treatment history, details of surgical intervention, postoperative recovery and complications, re-admission, histopathological report, disease recurrence, and further treatment history and follow-up period. The data obtained were analyzed and the results were tabulated and studied with the use of statistical software (IBM SPSS Statistics for Windows, Version 29.0. Armonk, NY: IBM Corporation).

## Results

A total of 13 patients had positive microscopic histologic margin (R1) among 172 patients who underwent gastrectomy of any type for GAC during the study period. Thus, the incidence of positive margins was 7.6% in our study. Out of them, nine patients were males and four were females. The median age was 48 years (range 27 to 69 years) (Table 1).

**Table 1:** Preoperative characteristics of patients

Characteristics	Number of patients (*N* = 13)
Median age (range)	49 years (27–69 years)
Sex ratio (M:F)	9:4
Presentation	
Abdominal pain Early satiety Melena Gastric outlet obstruction	7 4 3 5
Location of tumor	
Distal Proximal	12 1
Positive margin	
Distal only Proximal only Both proximal and distal	10 2 1
Neoadjuvant chemotherapy	
Yes No	3 10

The tumor location was in the distal stomach (including the antropyloric region and distal part of the body) in 92.3% of patients. Three patients received neoadjuvant chemotherapy (23.1%) while the remaining ten patients (76.9%) underwent upfront gastrectomy (Table 1).

In patients who underwent total gastrectomy, reconstruction was done using Roux-en-Y esophagojejunostomy while in patients who underwent subtotal or distal gastrectomy, reconstruction was done using loop gastrojejunostomy with Braun jejunojejunostomy. The median operative time was 220 min (190–390 min). The median duration of ICU stay was 2 days (1–5 days) (Table 2).

**Table 2:** Intra-operative and surgical characteristics

Characteristics	Number of patients (*N* = 13)
Type of gastrectomy	
Total Subtotal Distal Proximal	3 2 7 1
Median blood loss (ml)	100 ml (150–400 ml)
Median duration of surgery	220 min (190–390 min)

Two patients had post-operative complications. One patient had a burst abdomen and another patient had a duodenal blowout. Both of them expired on post-operative days 10 and 11, respectively. The rest of the 11 patients had an uneventful recovery.

The histopathological features of the surgical specimens are listed in Table 3. Eleven (84.6%) out of 13 patients had poorly differentiated carcinoma, and three among them had signet ring cell carcinoma. The distal margin was positive in 11 patients (84.6%) whereas the proximal margin was positive in 3 patients (23.1%). This included one patient who had both proximal and distal positive margins. Eight patients (61.5%) had T4 disease and four patients (30.8%) had T3 disease. Twelve (92.3%) patients had positive node disease, and out of which, six patients had extranodal extension (ENE).

**Table 3:** Histopathological characteristics of the specimen

Characteristics	Number of patients (*N* = 13)
Histology	
Adenocarcinoma Well-differentiated Poorly differentiated Signet-ring cell	13 2 11 3
Degree of differentiation	
Well differentiated Poorly differentiated	2 11
Margin positive	
Proximal Distal Both	2 10 1
T stage	
T2 T3 T4	1 4 8
Nodal status	
pN + /ypN + pN0/ypN0	12 1
Extranodal extension	
Yes No	6 7
Lympho-vascular invasion	
Yes No	8 5
Peri-neural invasion	
Yes No Not reported	8 3 2
Stage	
II III	3 10

Out of 11 patients, three patients received adjuvant chemoradiation while four patients received and completed adjuvant chemotherapy. Two patients defaulted adjuvant therapy after taking two cycles of chemotherapy and two patients did not receive any adjuvant therapy.

**Fig. 1:**
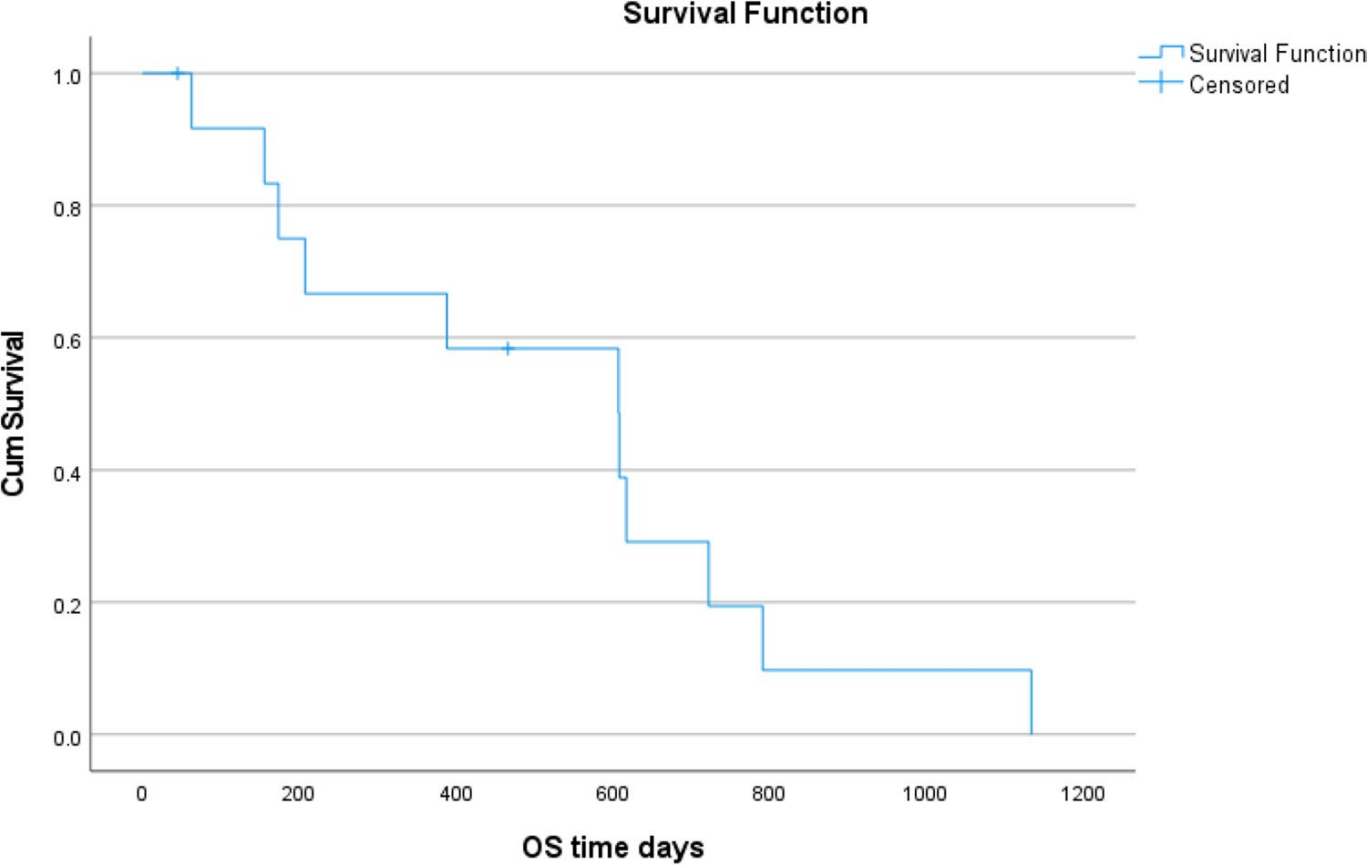
Overall survival curve

**Fig. 2:**
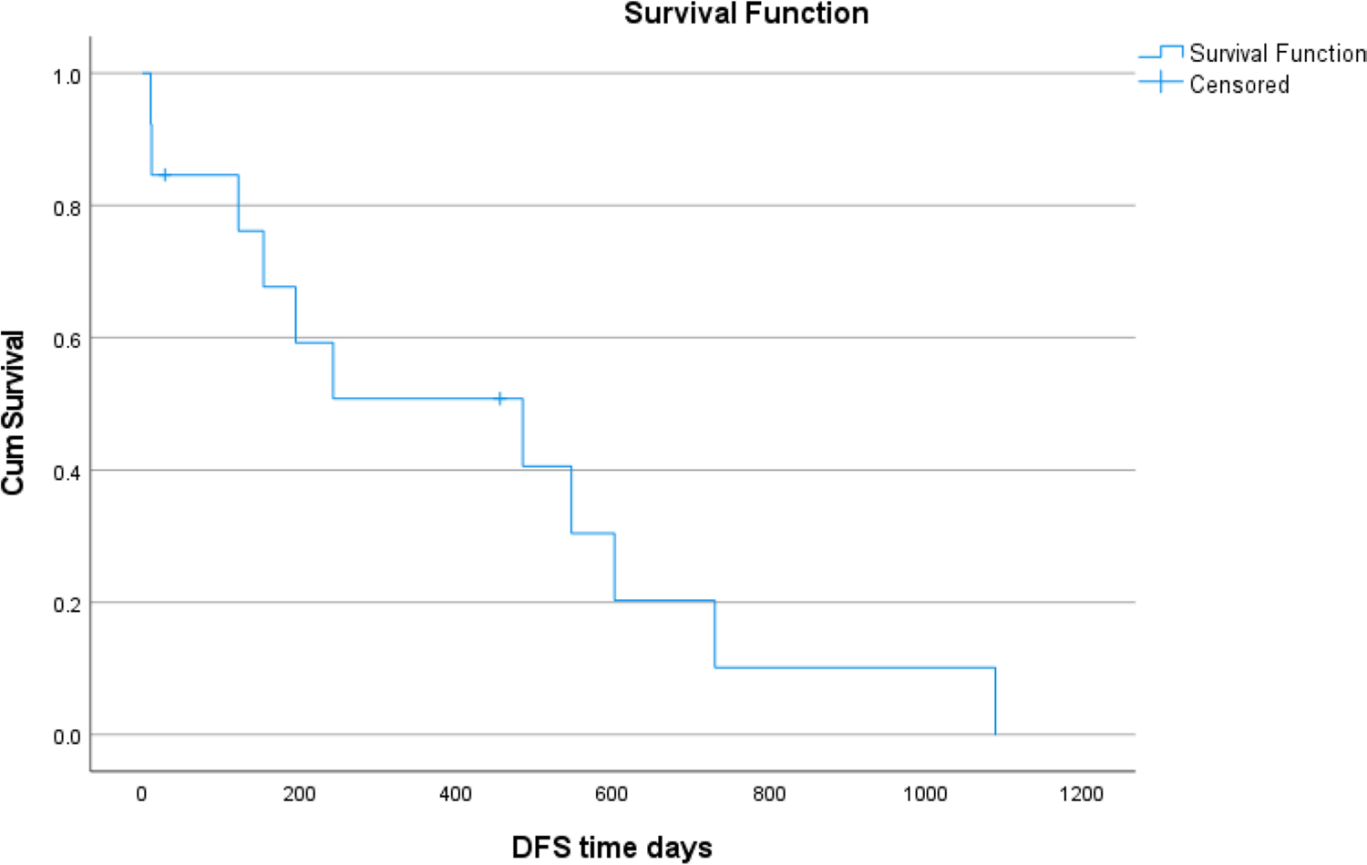
Disease-free survival curve

At the end of our study period, only 1 patient out of 13 patients was alive. Median disease-free survival (DFS) was 16.2 months (95% confidence interval 1.2 to 31.1 months). Median overall survival (OS) was 20.2 months (95% confidence interval 9.3 to 31.2 months). Median OS for those patients who had received any chemotherapy and chemoradiotherapy was 18 months (range was 7 to 25 months) and 35 months (range was 21 months to still not reached) (Figs. 1 and 2**).**

Out of the remaining 159 patients who did not have a documented margin-positive resection, complete data was retrievable for only 44 patients (27.7%). The median age of these patients was 60.5 years (range 26 to 85 years) and the male-to-female ratio was 3.4:1. The location of the tumor was mostly distal (79.5%), followed by the body (18.2%) and proximal (2.3%). Only 9.1% of these patients received neoadjuvant chemotherapy. The histopathological parameters of these patients were compared with those patients who had margin positivity, and it was found that a significant correlation was found only with N3 disease, the presence of perineural invasion (PNI), and poorly differentiated grade (Table 4).

**Table 4:** Comparison of histopathological parameters of patients with margin positivity and patients with margin negativity (complete data available subset)

Variable	Margin positive (*n* = 13)	Margin negative (*n* = 44) available data	*p* value	Interpretation
T1	0	8	0.096	Not significant
T2	1	8	0.362	Not significant
T3	4	14	0.944	Not significant
T4	8	14	0.536	Not significant
N1	4	11	0.674	Not significant
N2	1	9	0.289	Not significant
N3	7	10	0.031	Significant
LVI	8	23	0.555	Not significant
PNI	8	13	0.035	Significant
Well differentiated	2	19	0.067	Not significant
Moderately differentiated	0	9	0.075	Not significant
Poorly differentiated	8	9	0.045	Significant
Signet-ring cell	3	7	0.548	Not significant

*LVI* lymho-vascular invasion, *PNI* peri-neural invasion

## Discussion

Treatment options for locally advanced gastric cancer comprise multimodality therapy which consists of surgery and chemotherapy with the use of radiation as indicated. The Japanese Cancer Treatment Guidelines recommend surgery followed by adjuvant chemotherapy in early and locally advanced gastric cancer without bulky lymph nodes, whereas Western studies recommend peri-operative chemotherapy in locally advanced GC [3].

The incidence of positive margins following gastrectomy is variable ranging from 0.9 to 59% in various reported series, and its negative effect on survival outcomes has been demonstrated in many studies [5]. In a systematic review of 19,355 patients, it was shown that T3/T4 tumors, higher N status, diffuse-type gastric cancer, poorly differentiated tumors, lymphovascular invasion, and Bormann type 4 were predictive factors for positive resection margin [6]. In our study, among the patients who had microscopic positive margins, the frequency of T3/T4 disease was 92.3%, poorly differentiated histology was 84.6% and nodal positivity was 92.3% with extra-nodal extension in 50% of them, and lymphovascular invasion in 61.5% patients.

Intra-operative frozen section analysis can be a reliable method to decrease the incidence of positive margins in curative resection of gastric cancer. In a study of 110 patients with gastric cancer, it was reported that routine frozen section analysis of margins was able to detect positive margins in 14% of cases with no false negative result [7]. In another study of 377 patients, intra-operative frozen section analysis prevented positive resection margins. It was also found overall survival following re-resection of frozen section positive patients was similar to frozen section negative patients [8]. Shen et al. in their study of 66 patients with gastric adenocarcinoma of cardia observed that the accuracy, sensitivity, and specificity of an intra-operative frozen section were 97%, 77.8%, and 100%, respectively [9]. In our institute, routine examination of the resected margin with intra-operative frozen section analysis was not done previously.

The impact of a positive surgical margin is still not clear. Figueiredo et al. in their study showed that proximal resection margin was not a prognostic factor as long as R0 resection is achieved [4]. Postlewait et al. in their study of 162 patients with proximal gastric adenocarcinomas found that R1 resection was not associated with decreased survival but only increased local recurrence. However, on multivariate analysis, R1 resection was not independently associated with local recurrence [10]. Contrarily, Wang et al. [11] and Nagata et al. [12] found the microscopic positive resection margin an independent prognostic factor in both univariate and multivariate analysis across all stages. Bickenbach et al. [13] pointed out that R1 resection had no effect on survival in T3–T4 tumors and disease with greater than 3 positive lymph nodes. They observed a local recurrence rate of 16% in patients with R1 resection. Supporting this evidence, Cho et al. (14) and Cascinu et al. [15] found that the R1 resection margin is an independent negative predictor of survival only in N0 cases. In a systematic review of 19,992 patients, the R1 margin was associated with poorer overall survival [5].

There is a difference of opinion among medical practitioners regarding further management of patients with R1 resection. The reason is that there is no randomized controlled trial regarding the best management of patients with microscopic positive margins after gastrectomy. Though surgical re-resection to negative margins is a valid option, it is technically complex and is associated with high risks of mortality and morbidity. Moreover, recommendations in published literature regarding re-operation also vary. In a study which included 47 patients with margin-positive resection, Kim et al. showed that R1 margin status lost its prognostic value in multivariate analysis in all except in patients who had a low nodal burden of disease. They studied the patients who had positive margins on intraoperative frozen section analysis and compared the group of patients who had a re-resection to a negative margin with the group who left a residual resection line microscopically positive disease. Their finding was that the survival outcomes did not differ significantly between the two groups. However, in a subset analysis, when they compared the patients who had fewer than or equal to 5 positive nodes to those with more than five positive nodes, they found a significantly longer overall survival among the former subset. Thus, the presence of more than 5 positive nodes was found to be the primary determinant of survival and not the R1 status [16]. Aurello et al. [17] in their systematic review suggested that surgical re-excision should be considered for patients who have less than three metastatic lymph nodes. Cho et al. [14] also suggested re-excision for R1 cases in the absence of lymph node metastasis. Though there are no definite guidelines for the treatment of patients with R1 resection, it is best to present these cases in front of a multidisciplinary team and plan further treatment on a patient-to-patient basis. Probably patients with early-stage disease stand to benefit maximum from a re-resection. Other treatment options for this subset of patients are observation, chemotherapy, or chemoradiotherapy. None of the patients in our study had re-operation as all our patients were either T3–T4 or had lymph nodal metastasis. Patients receiving chemoradiotherapy had a better OS than patients receiving chemotherapy alone (median OS 35 months vs 18 months) but it is again subjected to bias. These patients were not fit for chemoradiotherapy after surgery as decided by our multidisciplinary team which was the reason for giving chemotherapy to them in the first place.

With the use of neoadjuvant therapy, the incidence of positive margins may be reduced as seen in our study that ten patients (77%) who underwent upfront surgery were margin-positive compared to three patients (23%) who received neoadjuvant therapy.

We understand that the retrospective nature of the study is a drawback. However, the long-term follow-up data is available for the patients with margin positivity. Another drawback is the availability of complete data for only 27.7% patients with a margin-negative resection. This being stated, we could compare the histopathological data of these patients with those having margin positivity.

## Conclusion

In conclusion, the incidence of positive margin in our study was 7.6%, and it is usually associated with advanced disease and aggressive tumor biology. Higher T stage and N stage, higher histologic grade, extranodal extension, PNI, and LVI were independent predictive factors of positive margins. Every effort should be made to achieve negative margins during initial resection. The intra-operative frozen section may be used to decrease the incidence of positive margins especially in early disease. The use of neoadjuvant therapy may reduce the incidence of positive margins.

## Data Availability

Data is available with the corresponding author.
